# Uncoupled mitochondria quickly shorten along their long axis to form indented spheroids, instead of rings, in a fission-independent manner

**DOI:** 10.1038/s41598-017-18582-6

**Published:** 2018-01-10

**Authors:** Yoshihiro Miyazono, Shingo Hirashima, Naotada Ishihara, Jingo Kusukawa, Kei-ichiro Nakamura, Keisuke Ohta

**Affiliations:** 1 Division Microscopic and Development Anatomy, Department of Anatomy Kurume University School of Medicine, Kurume, 830-0011 Japan; 2 Dental and Oral Medical Center, Kurume University School of Medicine, Kurume, 830-0011 Japan; 3 Department of Protein Biochemistry, Institute of Life Science, Kurume University, Kurume, 839-0864 Japan; 4 Advanced Imaging Research Center, Kurume University School of Medicine, Kurume, 830-0011 Japan

## Abstract

Loss of mitochondrial membrane potential (ΔΨm) triggers dramatic structural changes in mitochondria from a tubular to globular shape, referred to as mitochondrial fragmentation; the resulting globular mitochondria are called swelled or ring/doughnut mitochondria. We evaluated the early period of structural changes during the ΔΨm loss-induced transformation after carbonyl cyanide *m*-chlorophenyl hydrazine (CCCP) administration using a newly developed correlative microscopic method combined with fluorescence microscopic live imaging and volume electron microscopy. We found that most mitochondria changed from a tubular shape to a globular shape without fusion or fission and typically showed ring shapes within 10 min after CCCP exposure. In contrast, most ring mitochondria did not have a true through hole; rather, they had various indents, and 47% showed stomatocyte shapes with vase-shaped cavities, which is the most stable physical structure without any structural support if the long tubular shape shortens into a sphere. Our results suggested that loss of ΔΨm triggered collapse of mitochondrial structural support mechanisms.

## Introduction

Mitochondria are tubular-shaped, double membrane-bound organelles involved in various functions, including bioenergy production, apoptosis, autophagy, heme biosynthesis, oxygen sensing, and calcium homeostasis. On average, mitochondria have a size of 0.75–3 µm^2^, and their diameter and length vary considerably depending on the cell type or physiological state. Mitochondria can also form a network within the cell^1,2^. Emerging evidence has shown that mitochondria are dynamic organelles that undergo continuous fission and fusion, the balance of which not only controls their morphology and number, but also regulates mitochondrial function and distribution^3,4^. Although the relationship between mitochondrial shape and function is unclear, mitochondrial shapes are strictly determined by cells, organs, and species, and the elongation and fragmentation of mitochondria occur under different physiological or pathological conditions^5^. However, the role of the tubular shape of mitochondria and the mechanisms regulating mitochondrial shape have not been elucidated.

Recently, the relationship between morphological changes in mitochondria and reactive oxygen species (ROS) has been studied extensively^6^. Fragmentation of mitochondria is generally accepted as a typical reaction of cells to these stresses, and the fragmentation process has been reported to be caused by differential modulation of mitochondrial fission-fusion proteins^7^. A classic oxidative phosphorylation uncoupler, carbonyl cyanide *m*-chlorophenylhydrazone (CCCP), is frequently used to induce the opening of the permeability transition pore on the mitochondrial membrane, leading to the dissipation of mitochondrial membrane potential (ΔΨm)^8^. CCCP treatment also induces dramatic morphological changes in mitochondria. For example, a few hours after CCCP administration, the mitochondria change from an elongated network to small and globular structures scattered in the cell. Based on fluorescence microscopy, this event has been described as a type of mitochondrial fragmentation in which tubular mitochondria are thought to form vesicular shapes through a mechanism involving mitochondrial fission. The term “swelling” is also frequently used to describe dysfunctional mitochondria because they show increased permeability of the inner mitochondrial membrane, and the diameter of the vesicular structures also increases compared with the diameter of tubular structures^9^. Furthermore, ring-shaped (donut-shaped or toroidal) mitochondria have also been reported as a unique morphological features of mitochondrial dysfunction based on either electron microscopy or fluorescence microscopy. Ring-shaped mitochondria were first reported in the chick retinal pigment epithelium under continuous light by transmission electron microscopy (TEM)^10^. Similar ring-shaped mitochondria have also been observed in cultured cells under oxidative stress, e.g., following CCCP administration, by TEM^11^. Such cultured cells also show globular mitochondria with weak fluorescence in the centre and are referred to as ring-shaped mitochondria. In contrast, ring-shaped mitochondria have also been repored to form via self-fusion of tubular mitochondria, resulting in ring shapes as a reaction to the loss of mitochondrial membrane potential based on fluorescence microscopic live imaging of cultured cells after hypoxia^12^. However, it is unclear whether the decrease in mitochondrial membrane potential facilitates mitochondrial fission, fusion, or another mechanism. Furthermore, ring-shaped mitochondria observed by fluorescence microscopy are thought to have a through hole, and the presence of ring-shaped mitochondria may be associated with mitochondrial dysfunction. For example, ring-shaped mitochondria observed by electron microscopy were observed in synaptic terminals of a memory decline model in monkeys, suggesting that mitochondrial dysfunction is related to a disruption of important functions, including memory^13^.

The three-dimensional (3D) structures of CCCP-treated mitochondria in cultured cell have been characterised by electron tomography 3D reconstruction and electron microscopy using serial ultrathin sections. The spherical mitochondria frequently envelop the cytoplasm and other organelles and have a small orifice that connects the lumen to the cytosol^11^. This unique structure has been termed the “mitochondrial spheroid”. Formation of this mitochondrial spheroid is known to be induced by CCCP, although it is frequently used to trigger the mitophagy process, which is largely dependent on the expression of Parkin^14^. Moreover, formation of this spheroid requires the expression of mitofusins and is inhibited by ubiquitination and degradation of mitofusins via PARK2 E3 ubiquitin ligase. Therefore, mitochondrial spheroid formation may be another pathway for mitochondrial turnover^15^. Although structural transformation by CCCP administraton induces mitochondrial spheroid, the details of this transformation process, particularly during the acute period (within a few minutes after treatment) have not yet been elucidated.

While fluorescence microscopy can be used to visualise mitochondrial dynamics, visualisation of the detailed membrane organisation of mitochondria is quite difficult, even using super-resolution techniques. Additionally, because ring-shaped mitochondria observed by florescence microscopy have not been directly evaluated by electron microscopy, the relationship between mitochondrial spheroids and fluorescence microscopic ring-shaped mitochondria is unclear.

Here, we report the structural evaluation of the initial step of mitochondrial spheroid formation after the uncoupler-induced decline in mitochondrial membrane potential. To observe the detailed structure of the transforming mitochondria, we developed a new correlative light and electron microscopy (CLEM) method that combines light microscopic live-imaging and electron microscopic volume imaging using focused ion-beam scanning electron microscopy (FIB/SEM). FIB-SEM-based volume imaging is able to reconstruct the detailed mitochondrial structure, including cristae organisation, in a much wider area than that by the electron tomography method^16^; this volume CLEM can be used to visualise the detailed 3D membrane organisation of identical mitochondria observed in light microscopy. Using this method, we investigated the early stage of mitochondrial transformation after the induction of membrane potential loss by CCCP in mouse embryonic fibroblasts (MEFs) and HeLa cells. Our findings for the initial process of structural transformation after CCCP treatment provide important insights into the acute reaction of the cell to mitochondrial dysfunction.

## Results

### Effects of CCCP on mitochondria determined by fluorescence microscopy and TEM in HeLa cells and MEFs

**Figure 1:**
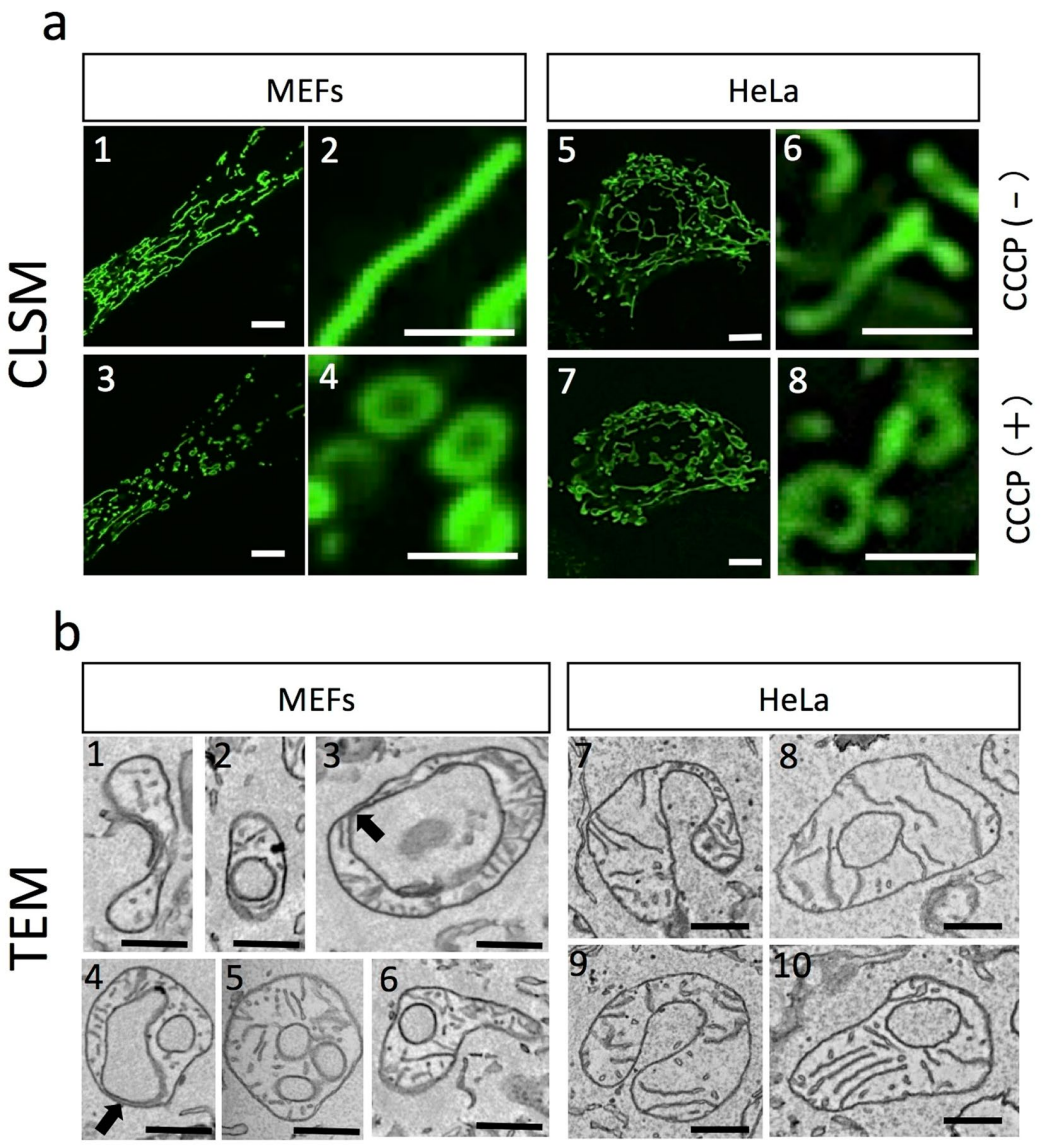
Confocal fluorescence and transmission electron microscopic (TEM) images of mitochondria in MEFs and HeLa cells 10 min after treatment with 10 μM CCCP. Confocal fluorescence images (**a**) of MEFs labelled with Su9 and HeLa cells labelled with GFP-PDHA showed typical tubular mitochondria before administration of CCCP (a1, a2, a5, and a6). After CCCP treatment, most mitochondria showed small globular shapes in lower-magnification images (a3 and a7) and ring shapes in higher-magnification images (a4, a8) in MEFs and HeLa cells. TEM observations of CCCP-treated cells (**b**) showed distinct U-, C-, and ring-shaped mitochondria in both cell types 10 min after administration. Each mitochondrion had an intact mitochondrial membrane and cristae structures. Some mitochondria had single or multiple lumina (b5 and b6). No differences were observed between cell lines. Scale bars, 0.5 μm (**b**) and 2 μm (**a**).

Structural changes in mitochondria after uncoupling were observed by fluorescence light microscopy and TEM in HeLa cells and MEFs, and we confirmed that most mitochondria transformed from the typical tubular shape into a spherical ring-like structure after uncoupler treatment in both cell lines (Fig. 1). *In situ* visualisation of mitochondrial membrane potential and ROS generation after CCCP administration with time-lapse observations showed an immediate decline in membrane potential (within a few seconds) and then a subsequent increase in ROS (~12 s) prior to the mitochondrial transformation (Supplementary Fig. S1). We observed the same cells before and 10 min after treatment with 10 μM CCCP, which induces uncoupling of mitochondria. Most mitochondria exhibited a change to the ring-shaped form after CCCP treatment. In MEFs treated with CCCP, tubular mitochondria became spherical (Fig. 1a1–a4), showing little or no green fluorescent protein (GFP) signal in the centre (Fig. 1a4,a8). This structure is referred to as a ring shape. Roundish mitochondria were similar in form and size, whereas the doughnut holes varied in size. Mitochondria in HeLa cells also showed shape changes from tubular to ring, as observed in MEFs, after CCCP treatment (Fig. 1a5–a8).

CCCP-treated mitochondria under TEM showed normal cristae, but no typical swelling of the matrix (Fig. 1b). In contrast, their shapes had unique features, such as a concave form with a flattened centre, e.g., a C- or U-shaped form (Fig. 1b1,b7,b9) and/or a ring-like shape (Fig. 1b2,b3,b8,b10). Such characteristics were observed in both MEFs (Fig. 1b1–b6) and HeLa cells (Fig. 1b7–b10). Ring-shaped mitochondria had a lumen that contained cytoplasm. The majority of mitochondria had a single lumen, although some had multiple lumina (Fig. 1b4,b5). Lumen sizes varied from a few hundred nanometres to a few micrometres. CCCP-treated mitochondria showed not only a rounded ring shape, but also composite features of both ring and tubular shapes, similar to the features of normal mitochondria (Fig. 1b6 and Supplementary Fig. S2). Notably, most CCCP-treated mitochondria had an extremely thin matrix space (Fig. 1b arrows and Supplementary Fig. S3). Even with such a thin matrix space, all mitochondria observed in this study had a continuous single matrix.

### Time-lapse observation of mitochondrial transformation after CCCP administration

**Figure 2:**
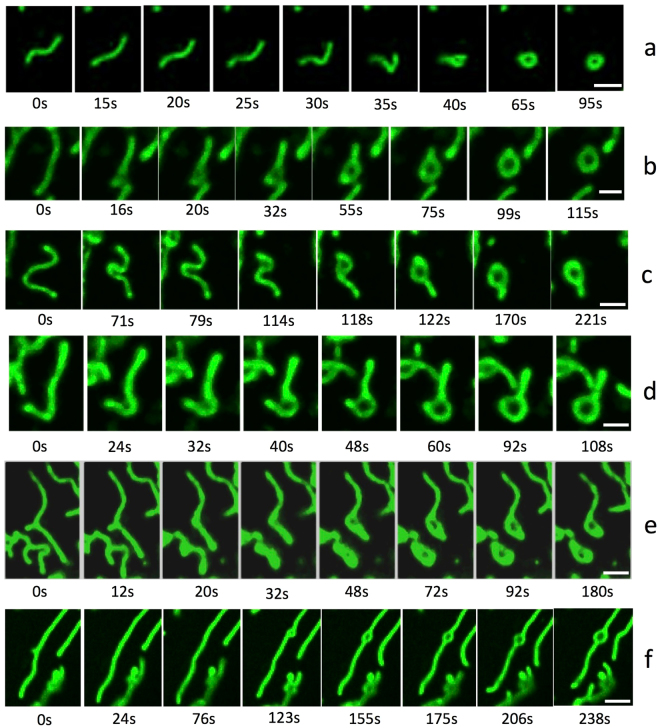
Time-lapse images of the mitochondrial transformation after CCCP administration observed by confocal microscopy. The time-lapse observations were started before administration, and the time elapsed after administration is indicated under each photograph. The series in (**a**) shows acute morphological changes in mitochondria from slender tubes to a ring-like structure within 1 min. The series in (**b**–**f**) shows that the ring-shape originated from the midpoint of mitochondrial tubes and that the diametre of the ring increased gradually. Scale bar, 2 μm.

**Figure 3:**
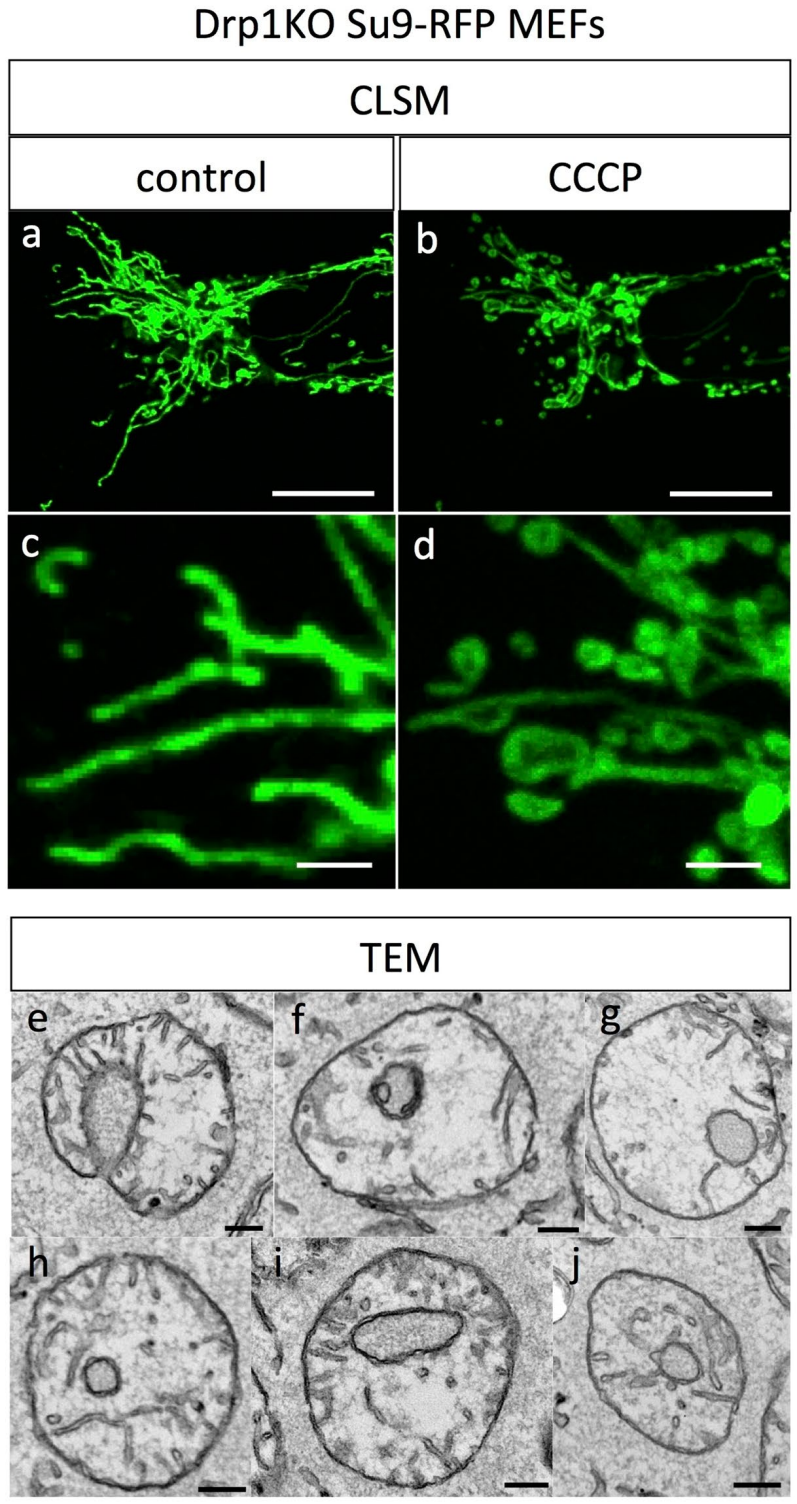
Confocal fluorescence and TEM images of mitochondria in Drp1-knockout Su9-RFP MEFs 10 min after treatment with 10 μM CCCP. Confocal fluorescence images (**a**,**b**,**c**,**d**) in Drp1-KO MEFs labelled with Su9 showed typical tubular mitochondria before administration of CCCP at lower and higher magnification (**a**,**c**). After CCCP treatment, most mitochondria showed small globular and ring shapes at a lower magnification (**b**) and higher magnification (**d**). TEM observations of CCCP-treated Drp1-KO MEFs (**e**–**j**) showed U-, C-, and ring-shaped mitochondria 10 min after administration. Scale bars,10 μm for a and b and 2 μm for c and d, and 0.2 μm for e–j.

Live imaging of mitochondria after CCCP treatment showed the process of shape transformation, which was completed within a few minutes (Fig. 2a–f). Most structural changes were initiated within tens of seconds after CCCP administration, and the transformation to the typical ring shape was completed within a few minutes. During this process, we focused on well-isolated mitochondria to avoid the possibility of overlapping of mitochondria, which would prevent us from distinguishing fusion versus overlap (arrows in Supplementary Movies S1–S6). In some cases, mitochondria showed bent shapes and seemed to shrink into a ring shape (Fig. 2a). Moreover, most mitochondria generated a ring-like structure by expansion of a part of the mitochondrion. This expansion was observed not only in the peripheral region (head or tail) of the mitochondria (Fig. 2b–e), but also in the central region (Fig. 2f). Interestingly, most of the ring shapes after CCCP treatment were generated from expansion of the mitochondria, rather than end-to-end fusion (Supplementary Movies S1–S6). From our observations, the diametre of the mitochondrial rings (expansion) increased gradually over time, and the tubular region tended to shorten during the process. Accordingly, long mitochondria tended to maintain their tubular shapes and gradually shortened as the ring region expanded like a balloon. In contrast, initially short mitochondria quickly shortened and formed a spheroidal shape. However, the maximum size of the ring was limited, and the expansion process reached a plateau within a few minutes. Notably, we did not observe mitochondrial fission or fusion during the rapid ring-shaped formation process at least 10 min after CCCP administration, even in completely isolated mitochondria. Because mitochondrial fission requires expression of dynamin-related protein 1 (Drp1), we further estimated the mitochondrial transformation in Drp1-knockout MEFs to evaluate whether spherical mitochondria formation required mitochondrial fission. In our results, similar ring-shape mitochondria were also observed, even in Drp1-knockout cells, under fluorescence microscopy and TEM (Fig. 3).

### Structure of CCCP-treated mitochondria, as determined using 3D volume CLEM combined with live imaging

**Figure 4:**
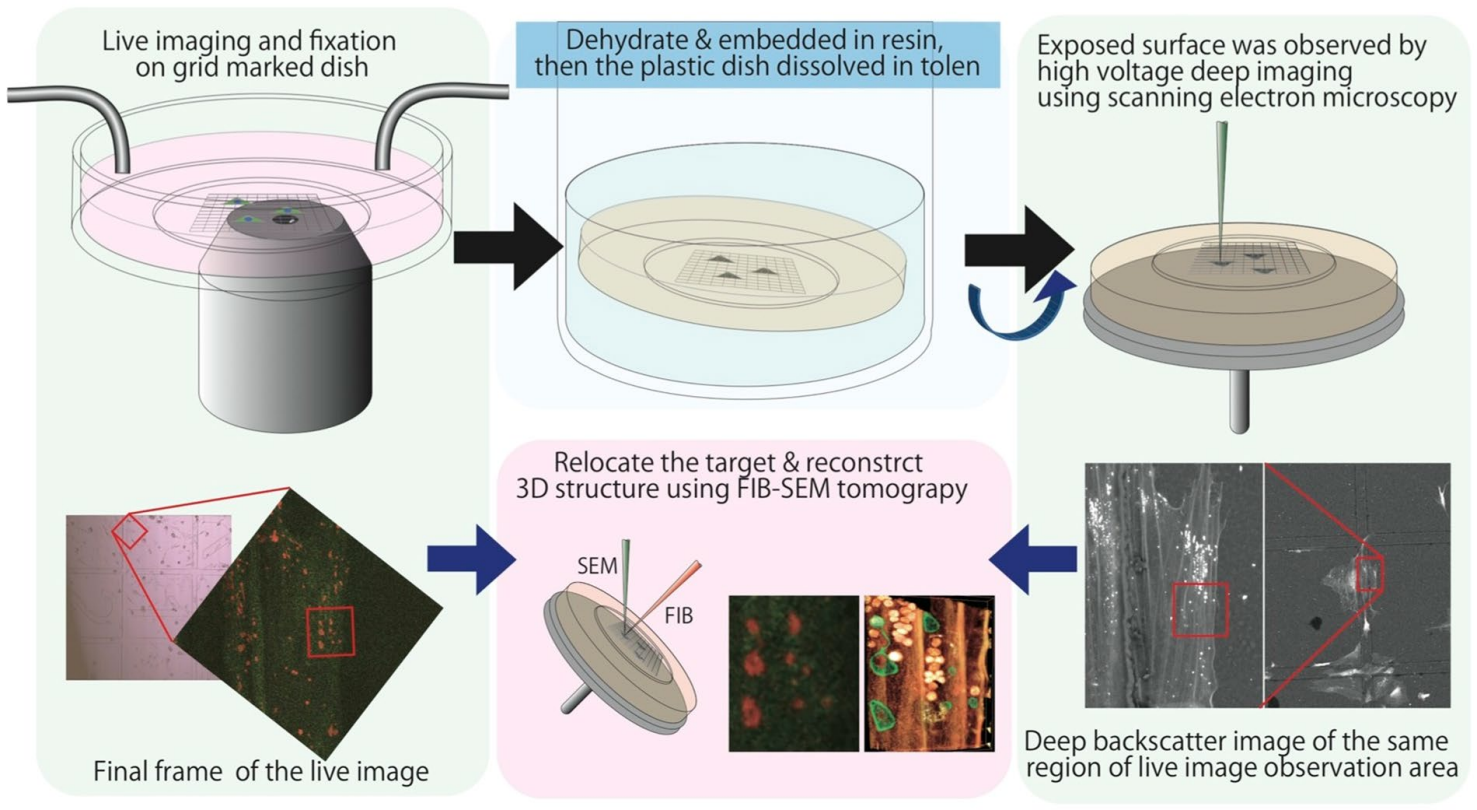
Schematic representation of 3D volume CLEM combined with live imaging. We developed a new technique to observe mitochondrial transformation after uncoupling in the acute stage. Cells were fixed during time lapse observation on a grid-marked 35-mm dish and then embedded in resin. The specimens were immersed in toluene to remove the dish. Subsequently, the cellular shape on the undersurface of resin-embedded cells was observed by SEM at a high acceleration voltage for relocation. The same region in light microscopy and electron microscopy analyses was reconstructed by FIB-SEM tomography at a high spatial resolution.

**Figure 5:**
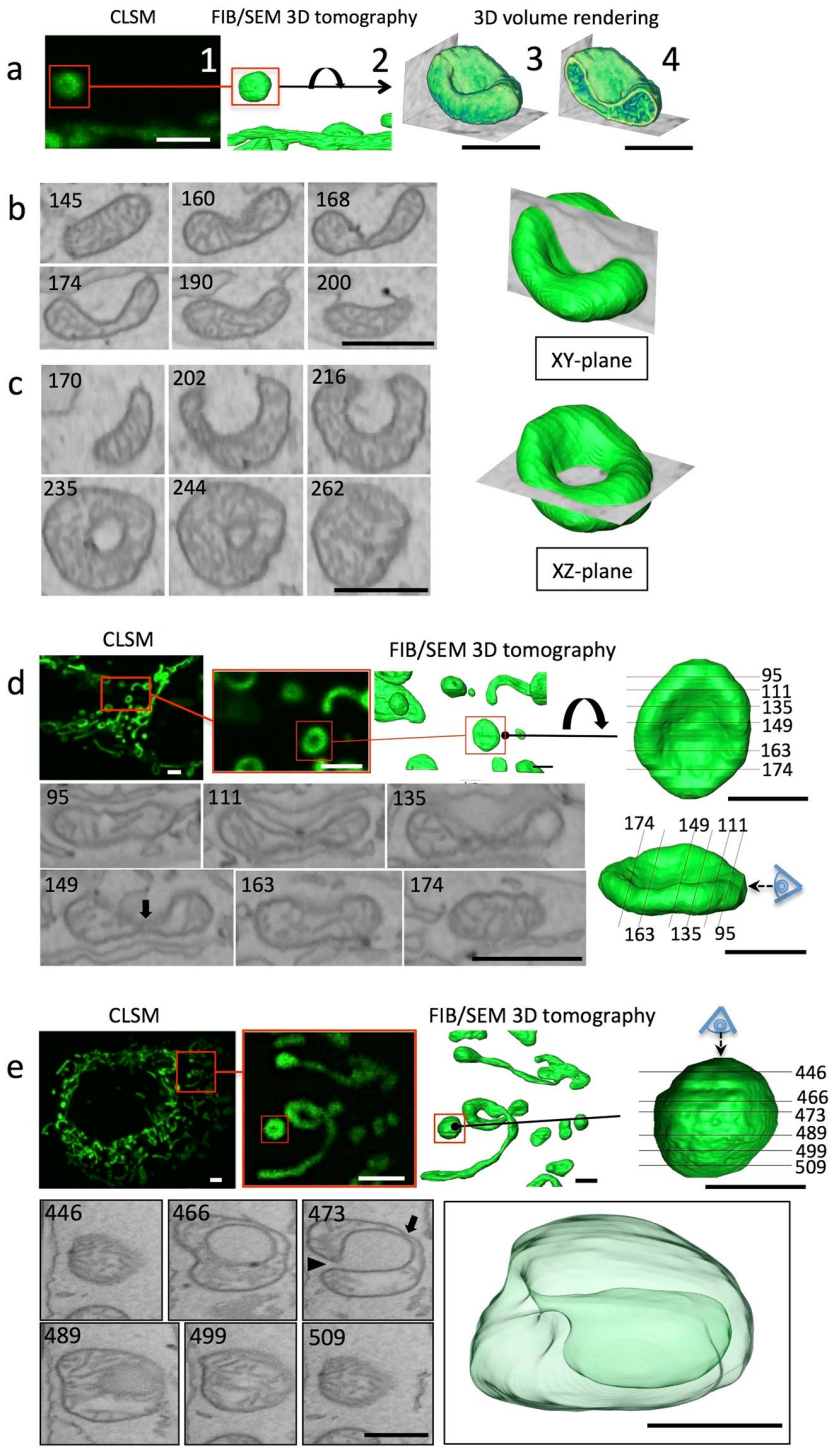
Correlative observation and interpretation of the structure of CCCP-treated mitochondria in MEFs using live imaging combined with 3D-CLEM. The last frame of live imaging of mitochondria after CCCP treatment showed the globular and ring-shaped mitochondria (red square in a1), which transformed from tube-shaped mitochondria. The whole frame of live imaging is shown in Supplemental Movie 7. Identical mitochondria were reconstructed by FIB-SEM tomography, and the same area is displayed in a2. The volume rendered view of the mitochondrion from the flipped direction shows the cup-shaped feature (a3), and some cristae were observed in cross-sections of the volume (a4). (**b**,**c**) Serial slice images of the volume data from different directions. Both discoidal mitochondria and vase-like stomatocyte-shaped mitochondria 10 min after CCCP treatment showed a ring shape in fluorescence microscopy (5d,5e). Two ring-shaped mitochondria (red squares) were selected under fluorescence microscopy, and identical mitochondria were reconstructed using the 3D-CLEM method based on FIB-SEM tomography. One showed a discoidal shape but did not have a through hole or invagination of the cytosol (**d**). The cross-section through the equator plane showed an erythrocyte-like biconcave shape and a very thin matrix at section 149, almost in contact with the opposite side of the membrane (arrow in section 149). The other (**e**) shows a large lumen connected to the cytosol through a small orifice (**e**, arrowhead). The translucent surface view shows the stomatocyte shape, also referred to as the mitochondrial spheroid structure (**e**), which also had a thin matrix portion (arrow in section 473) in the deepest region of the invagination. This shape was categorised as a “vase” in this work. Scale bar, 2 μm for CLSM images, 1 μm for others.

Structural changes in mitochondria after CCCP treatment were directly evaluated by CLEM combined with live imaging and FIB/SEM tomography (Figs 4 and 5). To relocate the same few-micron square region of cells by fluorescence microscopy and electron microscopy, we developed a new method (Fig. 4). During live imaging (4-s intervals), cells were fixed by the flow of the fixative, at which point mitochondrial motion was stopped within one interval. The locations of cells were recoded as grid numbers of the dish under fluorescence microscopy. Cells were then embedded in resin. As epoxy resin has resistance to toluene, the plastic dish was dissolved in the solvent. The remaining resin block, including cells, was then observed by SEM. The grid lines were cast in resin and easy to observe. Because high-voltage backscatter electron imaging enables visualisation of the cellular shape inside the resin^17^, we were able to easily relocate the light microscopic observation area. The volumes of the target areas were then reconstructed by FIB-SEM tomography with a 15-nm spatial resolution.

TEM observations (Fig. 1b and Supplementary Figs S2, S3) showed that CCCP-treated mitochondria had a typical ring form; however, we never observed an actual ring form in our 3D analysis, even for the mitochondria that showed ring-like shapes under light microscopy. Using this CLEM technique, we tracked a single mitochondrion by time-lapse imaging and observed a typical tubular-like mitochondrion that changed shape into a typical ring form (Fig. 5a, Supplementary Movie S7). Nevertheless, our 3D reconstruction showed that this mitochondrion had a concave shape with thick edges and a thin matrix in the centre, but did not have a through hole (Fig. 5a2,a3). Additionally, 3D volume rendering showed the formation of a mitochondrial membrane and the interior region composed of a cristae network and matrix area (Fig. 5a4). We generated computational slices of the 3D reconstructed mitochondrion (Fig. 5a3) at different angles (Fig. 5b,c). In serial cross-sections, the plane tangential to the edge of the mitochondrion (Fig. 5b *XY*-plane) showed a crescent shape (Fig. 5b section 174), whereas cross-sections through the horizontal plane (Fig. 5c *XZ*-plane) showed a ring shape from section 235 to section 244, similar to the TEM images shown in Fig. 1b.

Similarly, we analysed the 3D structure of additional ring-shaped mitochondria identified by light microscopy using the CLEM method (Fig. 5d,e). These ring-shaped mitochondria had various structural properties, including not only a concave shape, but also a discoidal (Fig. 5d) or vase-like shape (Fig. 5e) based on electron microscopy. The discoidal mitochondria had an oval biconcave form with dumbbell-shaped cross-sections, similar to the typical erythrocyte form (Fig. 5d sections 111, 135, and 149). The depressed centre was observed in the ring hole by light microscopy. Vase-shaped mitochondria were also observed with ring-shaped mitochondria by light microscopy (Fig. 5e). This type of mitochondria had invaginations in the outer membrane, with cytoplasm deep in the centre of the mitochondria and a narrow orifice (Fig. 5e section 473, arrowhead). The cross-sections of mitochondria frequently showed a ring shape or C shape with cytoplasmic lumen in two-dimensional images. Most 3D reconstructed mitochondria treated with CCCP showed a region of extremely thin matrix (approximately 50 nm; Fig. 5d section 149 and 5e section 473, arrows) with a poor cristae structure. Thus, ring-like mitochondria observed by light microscopy did not necessarily have a hole. Similar results were also obtained for HeLa cells and Drp1-knockout MEFs (Supplementary Figs S4, S5).

### 3D morphological characteristics of CCCP-treated mitochondria

**Figure 6:**
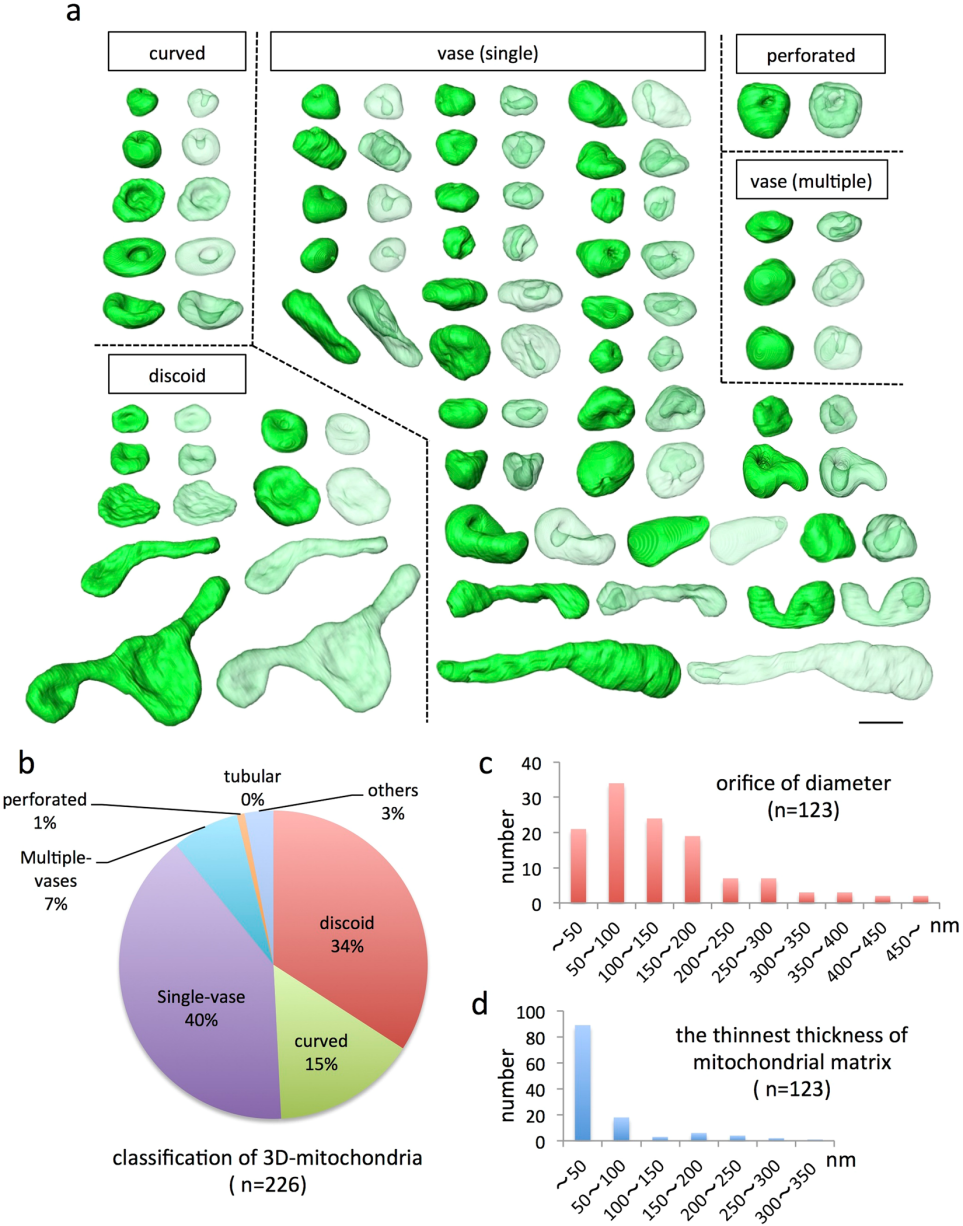
Mitochondrial shape 10 min after CCCP treatment evaluated by FIB-SEM tomography. Fifty-three randomly selected mitochondria (of a total of 226) from MEFs are shown (**a**). The shape of the mitochondria was categorised into seven groups, with adjacent images showing solid and translucent views of the surface rendering to observe invaginations. The proportion belonging to each group is shown in chart (**b**), and most mitochondria showed a vase shape (123/226). Structural characteristics of the orifice diameter (**c**) and the thinnest thickness of the mitochondrial matrix (**d**) in vase-shaped mitochondria are shown in histograms. Scale bar, 1 μm.

We reconstructed 226 mitochondria 10 min after CCCP administration. All mitochondrial shapes were extracted from the reconstructed volumes by segmentation (see Fig. 6a for examples). In this acute stage, many mitochondria had a cavity, i.e., an invagination of the mitochondrial outer membrane, generating a lumen. After careful observation of each mitochondrion, we classified them into seven groups (discoid, curved, single vase, multiple vase, perforated, and tubular; Fig. 6b), as described in detail in the Methods. Many transformed mitochondria in the acute phase had deep invaginations, creating a vase-like shape. Only 1% of mitochondria exhibited perforation and were topologically equivalent to the donut shape, but with a constricted region with a diametre of less than 100 nm. Additionally, 7% of mitochondria had multiple invaginations. The characteristics of vase-shaped mitochondria included narrow openings and a thin matrix between the bottom of the deep lumen and opposite side boundary membrane (Fig. 6c,d). The average diametre of the orifice was 154 nm (n = 123), and the median diametre was 120 nm. The inner boundary membrane of the bottom of the lumen was frequently in contact with the opposite side membrane (Supplementary Fig. S3), and the median matrix thickness was only 20 nm (Fig. 6d; n = 123), but some vase-shaped mitochondria had shallow cavities that did not come in contact with the opposite side.

### Invagination of the mitochondrial membrane pulled the cytoplasm and other organelles into the lumen

**Figure 7:**
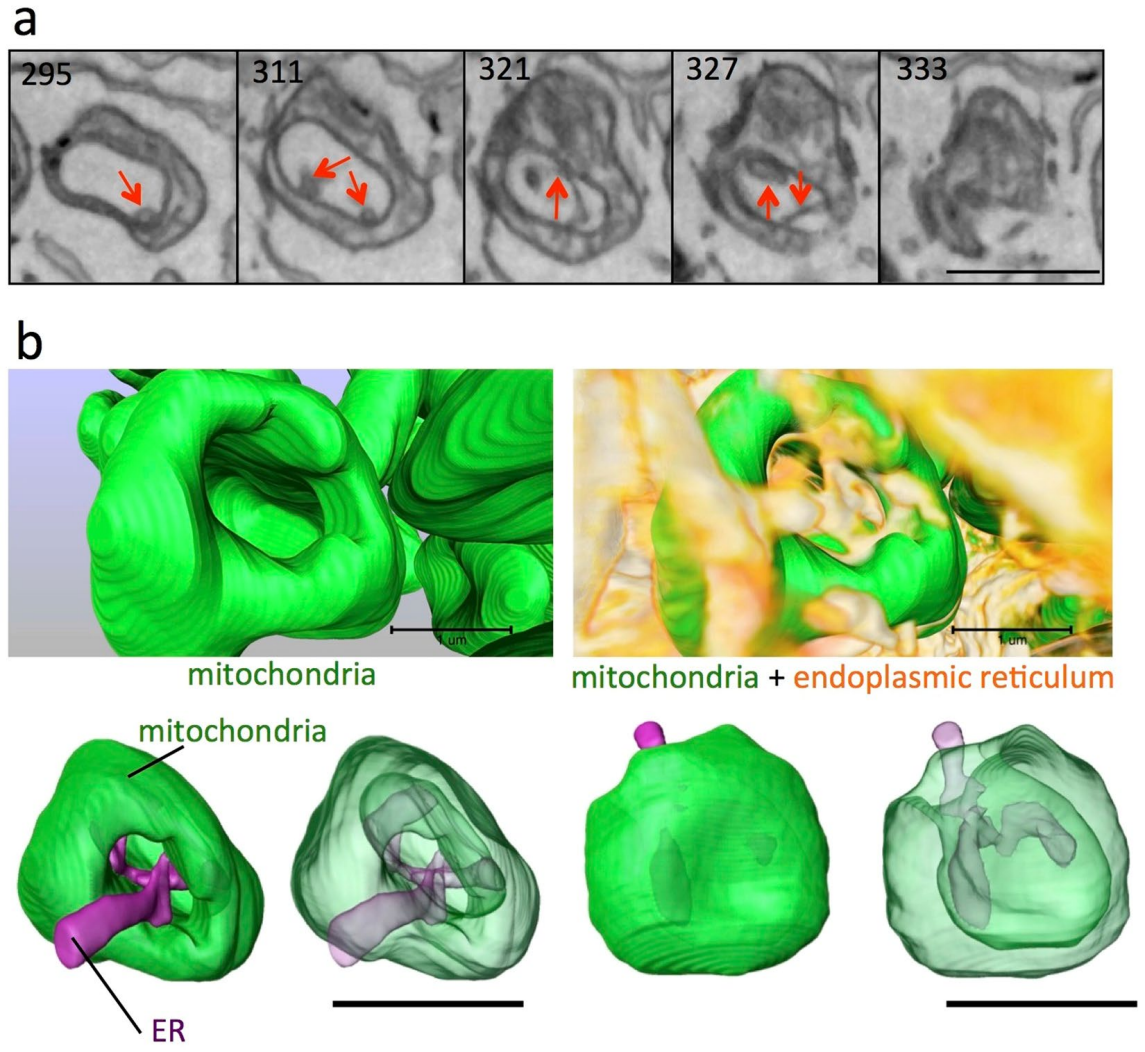
Three-dimensional interactions between the mitochondrial lumen and endoplasmic reticulum (ER) through the orifice in CCCP-treated vase-shaped mitochondria. In serial cross-sections by FIB-SEM (**a**), the endoplasmic reticulum occasionally appeared in the lumen of vase-shaped mitochondria induced by CCCP, and the ER frequently contacted the outer membrane of mitochondrial invaginations (arrows). The 3D reconstruction showed that the ER rose from the cytosol, entered the lumen of the mitochondria through the orifice, and spread into the lumen (**b**). Similar mitochondria with ER-associated lumina were observed in 14.6% of vase-shaped mitochondria. Scale bar, 1 μm.

We further examined the relationship between mitochondrial invagination and other organelles. The invaginated mitochondrial membrane pulled in cytoplasm with some tubular endoplasmic reticulum (ER) through a small orifice (Fig. 7a,b). The tubular ER inside the lumen seemed to make contact with the mitochondrial outer membrane (Fig. 7a, red arrow). ER included inside the mitochondrial lumen was present in 14.6% of vase-shaped mitochondria that we reconstructed. We also occasionally observed an incorporated lysosome in the invaginated lumina (Supplementary Fig. S6).

## Discussion

**Figure 8:**
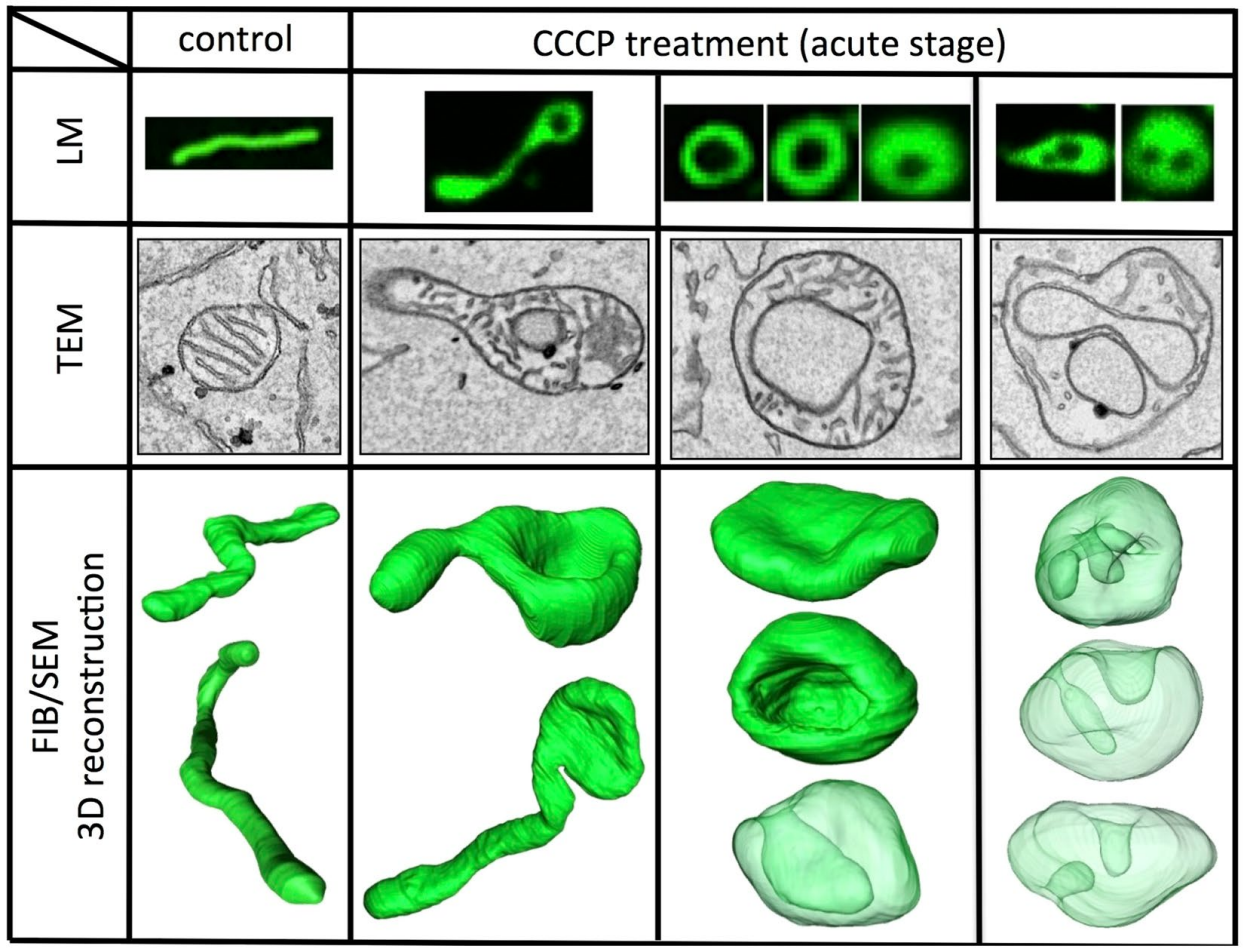
True shape of spherical mitochondria observed after CCCP treatment. Ring-shaped mitochondria were observed in fluorescent microscopy, as shown in the upper row, 10 min after CCCP treatment. Multiple void-like regions (or holes in rings) were sometimes observed. According to our observations, 1–3 lumina were present in TEM photomicrographs (middle row).

Loss of mitochondrial membrane potential induces dramatic structural changes from the typical tubular shape to an unusual spheroid shape with a thin matrix within a few minutes (Fig. 8; lower magnified 3D reconstruction images are shown in Supplementary Fig. S7). In this study, we characterised the 3D structural transformation process in this acute period for the first time and overcame the discrepancy between light microscopic and electron microscopic interpretations of mitochondrial structure. Our new correlative observation method between light and volume electron microscopy suggested that the transformation was a well-regulated process, resulting in the formation of relatively uniform round-shaped mitochondria with small cavities; these uniquely shaped mitochondria could form without mitochondrial fission or fusion within 10 min after the administration of an uncoupler.

Structural changes in the mitochondria are thought to be related to osmotic stress^18^ and oxidative damage in the cell^19^, although the significance of the structural transformation has not been established. Administration of an uncoupler, such as CCCP, FCCP, or valinomycin, increases proton or potassium-ionic permeability of the mitochondrial inner membrane and decreases the ΔΨm, which induces reactive oxygen species stress in the cell^8,11,20–23^. Such oxidative damage also induces mitochondrial spheroid formation^24^ and subsequent events, such as mitophagy and apoptosis. According to a previous study, uncoupler-induced structural changes are blocked by administration of antioxidants^19^. Such structural transformations are thought to be necessary for the next cellular adaptation process in response to mitochondrial dysfunction.

Our time-lapse live imaging showed that CCCP-induced mitochondrial transformation could proceed without active fission and fusion processes, although a decrease in ΔΨm is generally thought to facilitate mitochondrial fission and fragmentation from a long tubular-shaped mitochondrion to several small globular mitochondria. There is some controversy regarding the structural characteristics of mitochondria under oxidative stress. Although many light microscopy-based studies have suggested that uncoupling of mitochondria facilitates mitochondrial fission or fragmentation^25,26^, some studies have suggested that uncoupling facilitates their fusion^12^. Small spherical or globular mitochondria are usually observed in uncoupler-treated cells, typically a few hours after administration, and these reports have suggested that some mitochondria are thought to be a result of mitochondrial fission and fragmentation^27^. Uncoupler administration has also been reported to induce ring-shaped mitochondria, as demonstrated in more detail observations^12,21^. The centre of globular mitochondria usually shows weak fluorescence, and TEM observations have frequently shown ring-shaped mitochondria. Live imaging of this process has suggested that mitochondria under oxidative stress induce self-fusion to form an orbicularis structure^12^. Therefore, they concluded that structural transformation under stress is thought to be related to the mitochondrial fusion mechanism. However, our observations suggested another interpretation. Here, we showed that CCCP-treated mitochondria shrank from the end or middle of the tubular region during the transformation and then decreased in length. The site of shrinkage along its long axis showed a spherical bulb structure and an enlarged diametre during the process. At the same time, tubular-region mitochondria were incorporated into the spherical bulb and finally formed a simple globular spheroid within several minutes after administration. The diameters of globular mitochondria were slightly increased when compared with those of control tubular mitochondria. Similarly, Liu *et al*. reported that FCCP-treated or hypoxia-conditioned mitochondria showed increased diametres and volumes^21^. Consistent with previous reports, our observations also showed that the spheroids had a central region with weak fluorescence, and these could be referred to as ring-shaped mitochondria. Measurement of spheroid diametre in the FIB-SEM 3D reconstruction also showed an increase compared with the typical diametre of mitochondrial tubules, corresponding to the fluorescence microscopy analysis of Liu^21^. Notably, we seldom observed mitochondrial fission and fusion during the transformation, particularly during the acute stage. Additionally, similar spheroid formation was observed in Drp1-knockout cells. because Drp1 protein is an essential component of mitochondrial fission, acute spherical mitochondrial formation probably proceeded in a fission-independent manner.

Our 3D CLEM analysis further showed that the true shape of CCCP-treated ring-shaped mitochondria observed by fluorescence microscopy did not have a through hole and was identical to the biconcave or dumbbell-shaped mitochondria or spherical mitochondria with a unique cavity observed by electron microscopy. Classical electron microscopy has shown the existence of C-, U-, or O-shaped (ring-shaped) mitochondria in tissues in pathological states with oxidative stress, such as reoxygenation, hypoxia, and aging^10,13,28,29^. In contrast, fluorescence microscopy has also demonstrated the formation of unique ring-shaped mitochondria by oxidative stress, as discussed above^19,20,30^. Therefore, ring-shaped (toroidal) mitochondria are considered typical hallmarks of mitochondrial stress. Of course, we cannot exclude the possibility that some oxidative stress induces a true ring structure, as reported in synaptic boutons of ovariectomised aged monkeys based on TEM serial sections^13,31^. However, our CLEM observations clarified that acutely induced ring-shaped mitochondria after CCCP treatment identified by fluorescence microscopy rarely have through holes. Additionally, the weak fluorescence in the centre of mitochondria can be interpreted as the result of their cytoplasmic lumen or their thin mitochondrial matrix. In our 3D observations, many mitochondria were categorised as discoid, curved, and vase forms, all of which frequently had a thin matrix and a boundary membrane that was almost in contact with the opposite side at the deepest area of the dent. The thickness of the matrix was only 20 nm. Although we occasionally encountered mitochondria with a through hole to the opposite side that translated to the topological ring-shaped structure, our results also suggested that the acute response after loss of ΔΨm induced the formation of spherical mitochondria with a very thin matrix. Here, we examined the role of membrane topology. An indented sphere is topologically equivalent to a sphere, and a ring is categorised in a different topographical class, indicating that a ring cannot convert to an indented sphere easily. If fusion influences this transformation, a ring hole will emerge. Although the self-fusion process has been shown to be related to ring formation, we expect that is may be related to the long-term turnover of mitochondria as a constitutive process, which could result in generation of a through hole in mitochondria at low probability in the normal state and much higher probability in the long-term pathological state through fission and fusion. Therefore, we concluded that some mitochondria, particularly during the acute period, shortened along the long axis without fusion or fission, and this process could be another event facilitating fission/fusion-dependent mitochondrial transformation processes, such as fragmentation. Both of these processes may occur simultaneously in a cell after CCCP treatment.

Additionally, C-, U-, or O-shaped mitochondria observed by conventional TEM were simply cross-sections of these mitochondria. Notably, such characteristics may be difficult to visualise, even by TEM with serial sectioning or conventional 3D reconstruction methods. Our live-imaging volume-CLEM method is able to visualise a wide volume of light microscopic fields with a high spatial resolution and is advantageous to elucidate the true structure. This method is applicable to suborganellar mesoscale events, between the molecular and organellar levels.

The cavity structure on uncoupled mitochondria has been described by Ding^11,15^. Six hours after CCCP administration, a small orifice on the mitochondria with a narrow tube-like structure was found to connect the cytoplasm to the lumen by TEM serial sectioning and electron tomography. Although the observation period in the present study was earlier than that in the previous work, the structural characteristics of the cavity in our vase-shaped mitochondria were fundamentally similar to those of previous findings, and our results confirmed the previous hypothesis of mitochondrial spheroid formation. Because mitochondrial spheroids have been reported to decrese in loss of Parkin and mitofusin cell lines at a few hours after CCCP administration^24^, this uncoupling-dependent mitochondrial structural change is thought to be related to the mitochondrial degradation process. Additionally, we observed that this transformation frequently resulted in close proximity to the inner boundary membrane. Such arrangement of the two membranes suggests the existence of some interaction between the inner mitochondrial boundary membrane that has not been noted previously. However, we are considering the possibility that this structural change is a simple physical phenomenon resulting in a more stable structure.

The vase-shaped mitochondrial structures observed in this study resembled the shape of a stomatocyte; this shape is known to by physically stable, with a decreased volume:surface ratio compared with that of the sphere shape and the ability to transform from a sphere to a stomatocyte shape through an erythrocyte-like biconcave shape when the volume guradually decreases in the constant membrane area or transformation from complex structure to simple structure with the constant membrane area and the volume^32^. There are many ways to influence the balance of the volume:surface ratio of mitochondria after uncoupling, including sudden exchange of ions across the mitochondrial membrane, a change in the cristae organisation, or a change in the flip rate in the membrane component between the outer and inner mitochondrial membranes. However, the most simple way to change the ratio is a structural change from tubular to spherical. Some structural proteins may function to maintain the typical tubular mitochondrial structure, and uncoupling could affect the contact force between the membrane and structural proteins, thereby influencing membrane behaviour to induce structural transformation in the closed membrane shape to a much lower state of free energy. Elongated closed membranes not involved in structural support would show changes in structure from a tube shape to various globular shapes with small indents, including stomatocyte-like shapes, similar to red blood cells^33^. Various mitochondrial shapes observed in our study may be involved in the transformation to the final shape, e.g., mitochondrial spheroids^11^, and such variety also suggests that uncoupler-induced transformation occurs through a physical membrane mechanism to reach a structure with the lowest free energy.

In summary, our results contradicted the generally accepted interpretation of the CCCP-induced mitochondrial structural transformation process, such as fragmentation or swelling, based on precise morphological observations. As described above, the acute mitochondrial morphological changes caused by uncoupling may be related to physical phenomena. Therefore, we assumed that CCCP or other uncouplers would trigger the collapse of the mitochondrial system to maintain the tubular shape and shrink the mitochondria along the long axis into spherical structures, rather than facilitate fission and fusion during the early stages of uncoupling. Accordingly, our detailed estimation of structural transformation of mitochondria after uncoupling suggested that this transformation may be a useful model for further studies of the mechanisms mediating mitochondrial shape formation, although it is still unclear why mitochondria exhibit a long tubular shape. The reason for induction of structural transformation by loss of ΔΨm remains unclear; however, mitochondrial shrinkage along the long axis to a small spherical structure is favourable for subsequent events, such as mitophagy, that require other molecular mechanisms (Supplementary Fig. S8). The cavity structure is stable for at least 16 h in the absence of Parkin expression^24^. However, no cavity has been observed on the mitochondria in autophagosomes^34^. Therefore, we concluded that the mitochondrial fate after dysfunction may be determined by both an early physical process and later molecular processes (Supplementary Fig. S8). Further studies are required to clarify how uncoupling triggers mitochondrial transformation and how the transformation is related to the fate of dysfunctional mitochondria. Our findings are expected to improve our curent understanding of the morphological changes of mitochondria related to dysfunction.

## Materials and Methods

### Cell lines and culture

Su9-RFP MEFs, Drp1-knockout Su9-RFP MEFs^27^, and HeLa cells (ATCC CCL-2) were grown on glass-bottomed 35-mm culture dishes (Matsunami, Tokyo, Japan) for conventional observation and a μ-Dish 35 mm Grid-500 (Ibidi, Martinsried, Germany) for CLEM in Dulbecco’s modified Eagle’s medium (DMEM) with 10% foetal bovine serum (FBS) in an incubator with 5.0% CO_2_ at 37 °C. HeLa cells were transfected with CellLight Mitochondria-GFP or Mitochondria-RFP BacMam2.0 (Thermo Fisher Scientific, Waltham, MA, USA) to visualise mitochondria at 60–70% confluence in complete medium according to the manufacturer’s instructions.

### Live cell imaging and fixation

Cells were observed with a confocal laser scanning microscope (Fluoview, FV1000; Olympus, Tokyo, Japan). During sequential image acquisition, medium was changed to 10% FBS in DMEM with 10 μM CCCP (Wako, Osaka, Japan). Images were acquired at intervals of 4 s for approximately 10 min under the following conditions: objective, UPLSAPO 100× , NA 1.4 (Olympus); image size, 512 × 512 pixels; GFP fluorescence, excitation 473 nm, emission 488–585 nm; red fluorescent protein (RFP) fluorescence, excitation 559 nm, emission 570–670 nm. During imaging, fixative was added to the dish, and cells were further imaged for a few minutes. For CLEM, the location of the observation area was recorded along the grid. Thirty minutes before CCCP administration, prewarmed DMEM containing 250 nM TMRE (Nacali, Japan), or 2.5 μM CellROX green (Thermo Fisher Scientific) were added to the cells and incubated as described above. Medium containing TMRE or CellROX green was then removed, and normal growth medium was then added. As described above, CCCP was added during live-imaging observation.

### Sample preparation for electron microscopy

Ten minutes after CCCP administration, cells in time-lapse observation or conventional observation were fixed with half *Karnovsky* fixative (2% paraformaldehyde, 2.5% glutaraldehyde, 2 mM CaCl_2_ in 0.1 M cacodylate buffer [pH 7.3]) at room temperature (around 25 °C) and then placed on ice for 15 min. After washing five times with cacodylate buffer, the specimens were further fixed with 1.5% potassium ferrocyanide and 2% osmium tetroxide in 0.1 M cacodylate buffer at 4 °C for 30 min and then washed five more times with Milli-Q water. The specimens were then reacted with 1% thiocarbohydrazide solution at 60 °C for 1 h and washed five times with Milli-Q water. Subsequently, the specimens were further reacted with 2% osmium tetraoxide in Milli-Q water and washed five times with Milli-Q water. The specimens were then *en bloc* stained as follows. The specimens were first incubated with 4% uranyl acetate dissolved in distilled water overnight. After washing three times with Milli-Q water, specimens were immersed in Walton’s lead aspartate solution and then dehydrated with increasing concentrations of ethanol (20%, 50%, 70%, 80%, 90%, and twice in 100% for 5 min each), followed by infiltration with epoxy resin mixture (EPON812; TAAB, Reading, England). Samples were then polymerised for 72 h at 65 °C. After complete polymerisation, the resin blocks in plastic dishes were immersed in toluene to remove the dishes. The remaining resin-embedded specimens were placed in oven at 60 °C overnight.

### TEM observation

Resin-embedded specimens were trimmed into 0.5-mm squares and sectioned into 50-nm ultrathin sections using Ultracut E (Nissei, Tokyo, Japan) with a diamond-knife. The sections were then observed by H-7650 transmission electron microscopy (Hitachi High Technologies, Tokyo, Japan) without further staining.

### FIB-SEM tomography

The 3D ultrastructure of the cells was analysed by FIB/SEM tomography^16^. For conventional observation (without correlations), cover glasses were removed by heating the specimen on a hot plate (105 °C). The resin blocks were then mounted on aluminium stubs, adhered using silver paste (Dotite D550; Fujikura Kasei, Tokyo, Japan), and coated with evaporated carbon. The specimen was set in FIB-SEM machinery (Quanta 3D FEG; FEI, Eindhoven, The Netherlands) and observed by SEM at 7–30 kV using a backscatter detector; the site for reconstruction was thus determined. For CLEM, the grid and cellular shape were easily recognised under this condition. Serial images from the block were acquired as described previously^16^ under the following conditions. Milling was performed with a gallium ion beam at 30 kV and a beam current of 1 nA. The slice pitch was set to 15 nm/step. Images were acquired at a landing energy of 2.5 keV. The milling and imaging cycle was repeated 1,000 times. Additional acquisition parameters were as follows: beam current = 51 pA, dwell time = 6 µs/pixel, image size = 2048 × 1768 pixels, and pixel size = 4.8 nm/pixel. The resulting image stacks were analysed using Avizo 8.1 (FEI, Burlington, MA, USA). Mitochondrial shapes were traced using the semimanual technique^16^ implemented in Avizo 8.1.

### Mitochondrial classification

Reconstructed mitochondria (n = 226) after CCCP treatment were classified into seven groups (discoid, curved, single vase, multiple vase, perforated, and tubular). Tubular mitochondria were the conventional type, usually observed in control cells. Discoid mitochondria included biconcave shapes. Curved mitochondria included cup-shaped mitochondria without an overhang at the edge of invagination. Vase-shaped mitochondria had an overhang at the entrance of invagination, which could be called an orifice, with an expanding lumen. Furthermore, vase-shaped mitochondria were classified into two groups (single and multiple) according to the number of invaginations. Perforated mitochondria were distinguished by topological characteristics, including the presence of a hole in true ring-shaped or toroidal mitochondria.

## Electronic supplementary material

supplementary figures and legends

supplementary movie 1

supplementary movie 2

supplementary movie 3

supplementary movie 4

supplementary movie 5

supplementary movie 6

supplementary movie 7

## References

[1] Rafelski SM, Marshall WF (2008). Building the cell: design principles of cellular architecture. Nat. Rev. Mol. Cell Biol..

[2] Bereiter-Hahn J (1990). Behavior of mitochondria in the living cell. Int. Rev. Cytol..

[3] Polyakov VY, Soukhomlinova MY, Fais D (2003). Fusion, fragmentation, and fission of mitochondria. Biochemistry (Mosc.).

[4] Chan DC (2006). Mitochondrial fusion and fission in mammals. Annu. Rev. Cell Dev. Biol..

[5] Bleazard W (1999). The dynamin-related GTPase Dnm1 regulates mitochondrial fission in yeast. Nat. Cell Biol..

[6] Wang Y, Nartiss Y, Steipe B, McQuibban GA, Kim PK (2012). ROS-induced mitochondrial depolarization initiates PARK2/PARKIN-dependent mitochondrial degradation by autophagy. Autophagy.

[7] Wu S, Zhou F, Zhang Z, Xing D (2011). Mitochondrial oxidative stress causes mitochondrial fragmentation via differential modulation of mitochondrial fission-fusion proteins. FEBS J..

[8] Ganote CE, Armstrong SC (2003). Effects of CCCP-induced mitochondrial uncoupling and cyclosporin A on cell volume, cell injury and preconditioning protection of isolated rabbit cardiomyocytes. J. Mol. Cell Cardiol..

[9] Nowikovsky K, Schweyen RJ, Bernardi P (2009). Pathophysiology of mitochondrial volume homeostasis: potassium transport and permeability transition. Biochim. Biophys. Acta.

[10] Lauber JK (1982). Retinal pigment epithelium: ring mitochondria and lesions induced by continuous light. Curr. Eye Res..

[11] Ding WX (2012). Electron microscopic analysis of a spherical mitochondrial structure. J. Biol. Chem..

[12] Long Q (2015). Modeling of mitochondrial donut formation. Biophys. J..

[13] Hara Y (2016). Estrogen restores multisynaptic boutons in the dorsolateral prefrontal cortex while promoting working memory in aged rhesus monkeys. J. Neurosci..

[14] Narendra D, Tanaka A, Suen DF, Youle RJ (2008). Parkin is recruited selectively to impaired mitochondria and promotes their autophagy. J. Cell Biol..

[15] Yin XM, Ding WX (2013). The reciprocal roles of PARK2 and mitofusins in mitophagy and mitochondrial spheroid formation. Autophagy.

[16] Ohta K, Okayama S, Togo A, Nakamura K (2014). Three-dimensional organization of the endoplasmic reticulum membrane around the mitochondrial constriction site in mammalian cells revealed by using focused-ion beam tomography. Microscopy (Oxf.).

[17] Richards RG, Gwynn IA (1995). Backscattered electron imaging of the undersurface of resin-embedded cells by field-emission scanning electron microscopy. J. Microsc..

[18] Nowikovsky K, Reipert S, Devenish RJ, Schweyen RJ (2007). Mdm38 protein depletion causes loss of mitochondrial K+/H+ exchange activity, osmotic swelling and mitophagy. Cell Death Differ..

[19] Ahmad T (2013). Computational classification of mitochondrial shapes reflects stress and redox state. Cell Death Dis..

[20] Safiulina D, Veksler V, Zharkovsky A, Kaasik A (2006). Loss of mitochondrial membrane potential is associated with increase in mitochondrial volume: physiological role in neurones. J. Cell Physiol..

[21] Liu X, Hajnóczky G (2011). Altered fusion dynamics underlie unique morphological changes in mitochondria during hypoxia-reoxygenation stress. Cell Death Differ..

[22] Polygalova OO, Ponomareva AA (2010). [Protonophores as inducers of energy dependent changes in the ultrastructure of wheat root cells mitochondria]. Tsitologiia.

[23] Itami N, Shiratsuki S, Shirasuna K, Kuwayama T, Iwata H (2015). Mitochondrial biogenesis and degradation are induced by CCCP treatment of porcine oocytes. Reproduction.

[24] Ding WX (2012). Parkin and mitofusins reciprocally regulate mitophagy and mitochondrial spheroid formation. J. Biol. Chem..

[25] Frank S (2001). The role of dynamin-related protein 1, a mediator of mitochondrial fission, in apoptosis. Dev. Cell.

[26] Skulachev VP (2004). Thread-grain transition of mitochondrial reticulum as a step of mitoptosis and apoptosis. Mol. Cell Biochem..

[27] Ishihara N, Jofuku A, Eura Y, Mihara K (2003). Regulation of mitochondrial morphology by membrane potential, and DRP1-dependent division and FZO1-dependent fusion reaction in mammalian cells. Biochem. Biophys. Res. Commun..

[28] Stephens RJ, Bils RF (1965). An atypical mitochondrial form in normal rat liver. J. Cell Biol..

[29] Kiessling KH, Tobe U (1964). Degeneration of liver mitochondria in rats after prolonged alcohol consumption. Exp. Cell Res..

[30] Sharma J, Johnston MV, Hossain MA (2014). Sex differences in mitochondrial biogenesis determine neuronal death and survival in response to oxygen glucose deprivation and reoxygenation. BMC Neurosci..

[31] Hara Y (2014). Presynaptic mitochondrial morphology in monkey prefrontal cortex correlates with working memory and is improved with estrogen treatment. Proc. Natl. Acad. Sci. USA.

[32] Salva R (2013). Polymersome shape transformation at the nanoscale. ACS Nano.

[33] Lim HWG, Wortis M, Mukhopadhyay R (2002). Stomatocyte-discocyte-echinocyte sequence of the human red blood cell: evidence for the bilayer- couple hypothesis from membrane mechanics. Proc. Natl. Acad. Sci. USA.

[34] Yoshii SR, Kishi C, Ishihara N, Mizushima N (2011). Parkin mediates proteasome-dependent protein degradation and rupture of the outer mitochondrial membrane. J. Biol. Chem..

