# Supplementary material for: Uncoupled mitochondria quickly shorten along their long axis to form indented spheroids, instead of rings, in a fission-independent manner
Source: Sci Rep. 2018 Jan 10;8:350. doi: 10.1038/s41598-017-18582-6 (PMC5762872; doi:10.1038/s41598-017-18582-6)

Uncoupled mitochondria quickly shorten along their long axis to form indented spheroids, instead of rings, in a fission-independent manner

Yoshihiro Miyazono[1,2], Shingo Hirashima[1], Naotada Ishihara[3], Jingo Kusukawa[2], and Kei-ichiro Nakamura[1], Keisuke Ohta[1,4]

[1]Division Microscopic and Development Anatomy, Department of Anatomy Kurume University School of Medicine, Kurume 830-0011, Japan

[2]Dental and Oral Medical Center, Kurume University School of Medicine, Kurume 830-0011, Japan

[3]Department of Protein Biochemistry, Institute of Life Science, Kurume University, Kurume 839-0864, Japan

[4]Advanced Imaging Research Center, Kurume University School of Medicine, Kurume 830-0011, Japan

Correspondence should be addressed to K.O. (E-mail:kohta@med.kurume-u.ac.jp)

Supplementary Figure Legends

Supplementary Fig. S1. Sequential images of MEFs stained with tetramethyl rhodamine ethyl ester (TMRE) or CellROX green after CCCP administration.

(a) Mitochondrial membrane potential was assessed by TMRE. After CCCP administration, the fluorescence intensity was gradually reduced. (b) Reactive oxygen species in cells were assessed by CellROX green, which binds to oxidized DNA. We observed the oxidative status in cells after CCCP administration. The fluorescence intensity was gradually increased within 10 min. After 30 min, nuclei were strongly stained green, and mitochondria became more ring-shaped.

Supplementary Fig. S2. TEM images of mitochondria showed a tubular structure (arrows) with a bulb region in the lumen (arrowheads) in both MEFs and HeLa cells 10 min after treatment with 10 µM CCCP. These micrographs show mitochondria composed of a ring part and a tubular part. Scale bars, 0.5 µm for MEFs and 1 µm for HeLa cells.

Supplementary Fig. S3. TEM images of mitochondria in MEFs and HeLa cells 10 min after treatment with 10 µM CCCP. Mitochondria showing C, U, and O shapes had extremely thin matrix spaces within the middle of the structure for the U- and C-shaped mitochondria and located to one side for the O-shaped mitochondria. Scale bars, 0.5 µm.

Supplementary Fig. S4. Structure of CCCP-treated mitochondria in HeLa cells, as determined by live imaging combined with 3D-CLEM. Most mitochondria showed changes in the ring shape from their original tube shape 10 min after CCCP treatment. Identical areas observed by fluorescent microscopy (red square) were reconstructed and displayed by 3D-CLEM. Mitochondria in HeLa cells also showed various shapes, similar to those in MEFs, with discoid, curved, and vase shapes observed in 3D images.

Supplementary Fig. S5. The structure of CCCP-treated mitochondria in Drp1-knockout MEFs, as determined by live imaging combined with 3D-CLEM. Most mitochondria showed changes in the ring shape from their tubular shape within 10 min after CCCP treatment. Identical areas (a–f) observed by fluorescent microscopy (red squares) were reconstructed and displayed by 3D-CLEM. Mitochondria in Drp1-knockout MEFs also showed indented shapes, similar to those in wild-type MEFs and HeLa cells, without through holes in 3D reconstruction images.

Supplementary Fig. S6. CCCP-treated mitochondrion occasionally incorporated lysosomes into their invaginations. Serially sectioned images (left) and 3D surface rendering views (right) are displayed in different directions from the FIB-SEM tomography reconstruction data. Scale bars, 1 μm.

Supplementary Fig. S7. Three-dimensional distribution of mitochondria in control and CCCP-treated MEFs obtained using FIB-SEM. Mitochondria had tubular shapes in the control group, but spherical shapes 10 min after treatment with 10 μM CCCP. Scale bars, 5 μm.

Supplementary Fig. S8. Model of uncoupling-induced mitochondrial transformation. Elongated tube-like mitochondria shrank into a spherical structure with a small cavity (stomatocyte shape) using various shapes with a thin matrix by a physical process triggered by a loss of $\Delta\Psi m$. This process was completed within a few min. The final structure was considered a stomatocyte shape and was stable when the volume:surface ratio was low. After transformation, the mitochondria underwent subsequent processes based on molecular mechanisms.

Supplementary Movie Legends

Supplementary Movies S1–S6. Sequential movies of mitochondrial transformation by CCCP treatment. These CLSM live images show the structural transformation of mitochondria after CCCP treatment within a few minutes (see also Fig. 2).

Supplementary Movie S7. Time-lapse movie of CCCP-induced mitochondrial transformation shown in Figure 5a. The last frame of this movie is depicted in Fig.5a. CLEM was performed to track the single mitochondrion.

Supplemental Figure S1

a. mitochondrial membrane potential

CCCP administration

TMRE

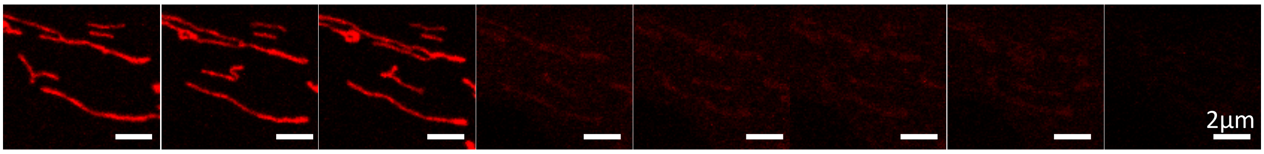

0 S 20 S 80 S 92 S 160 S 200 S 360 S 560 S

b. reactive oxygen species in cell

CCCP administration

Cell ROX

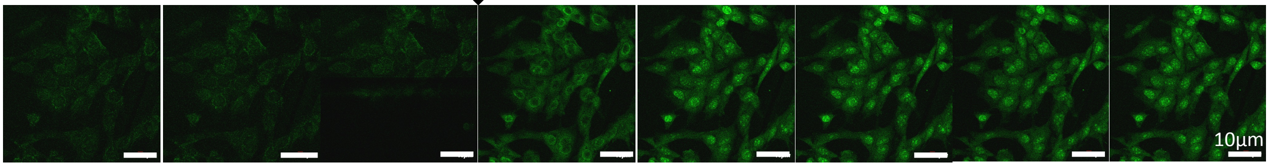

0 S 20 S 80 S 92 S 160 S 200 S 360 S 560 S

CellROX green
Su9-RFP mitochondria

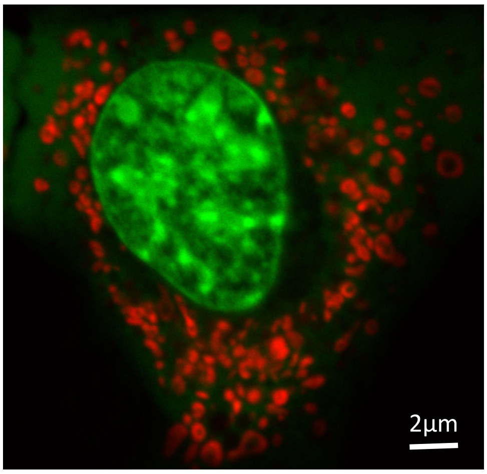

30 min

Supplementary Figure S2

MEFs

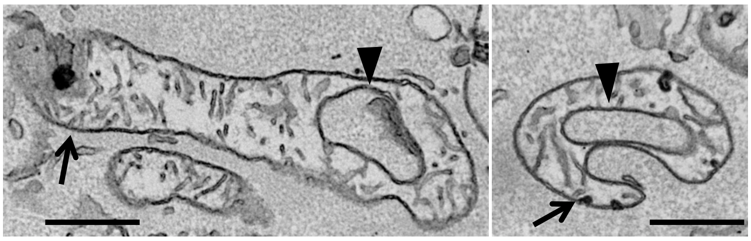

HeLa cell

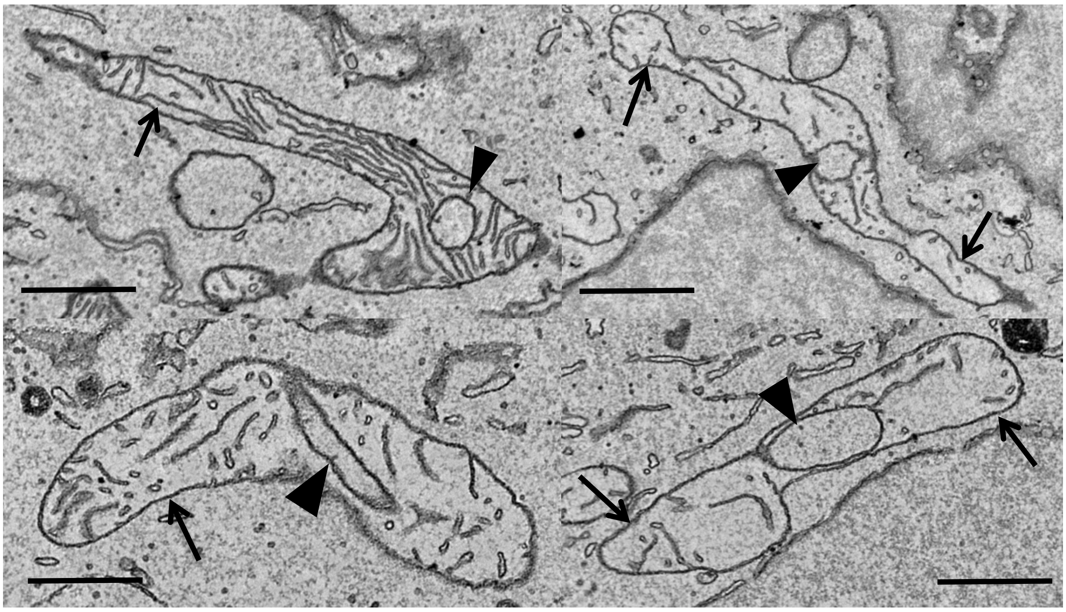

Supplementary Figure S3

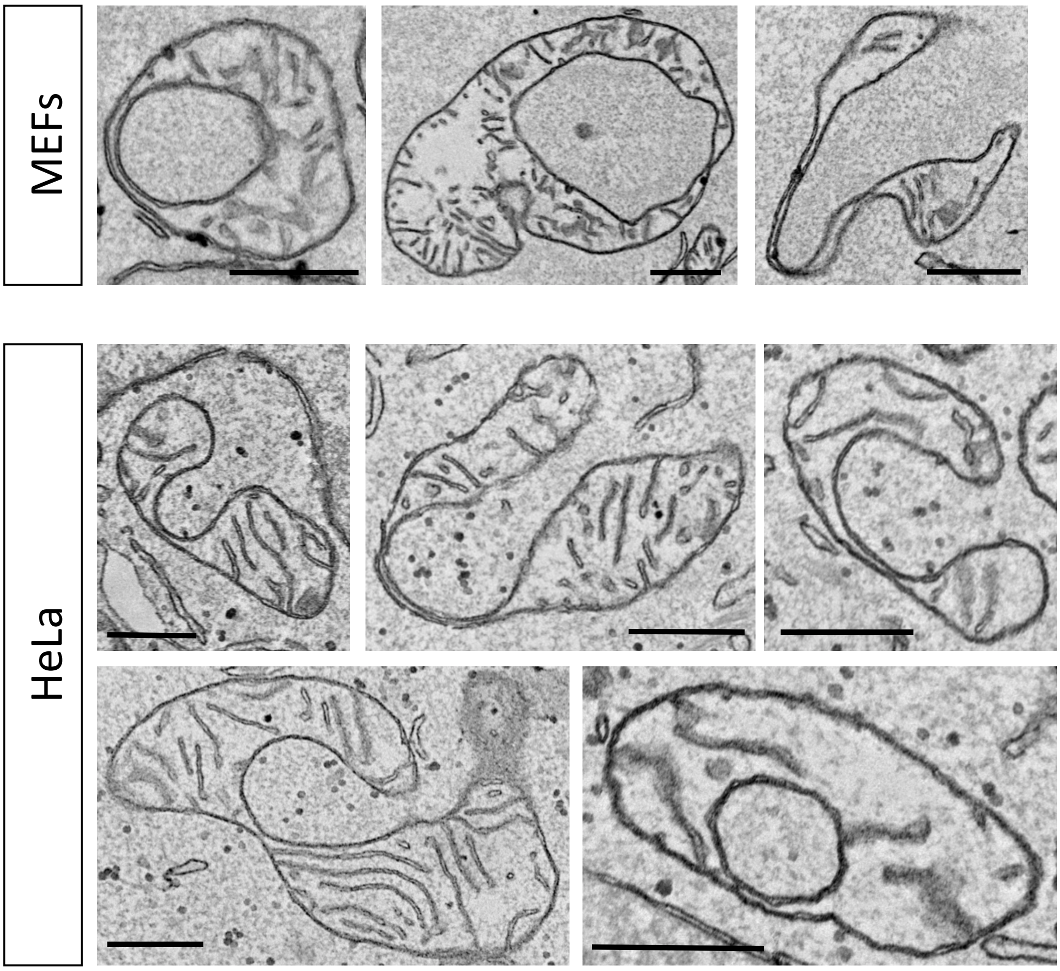

Supplementary Figure S4

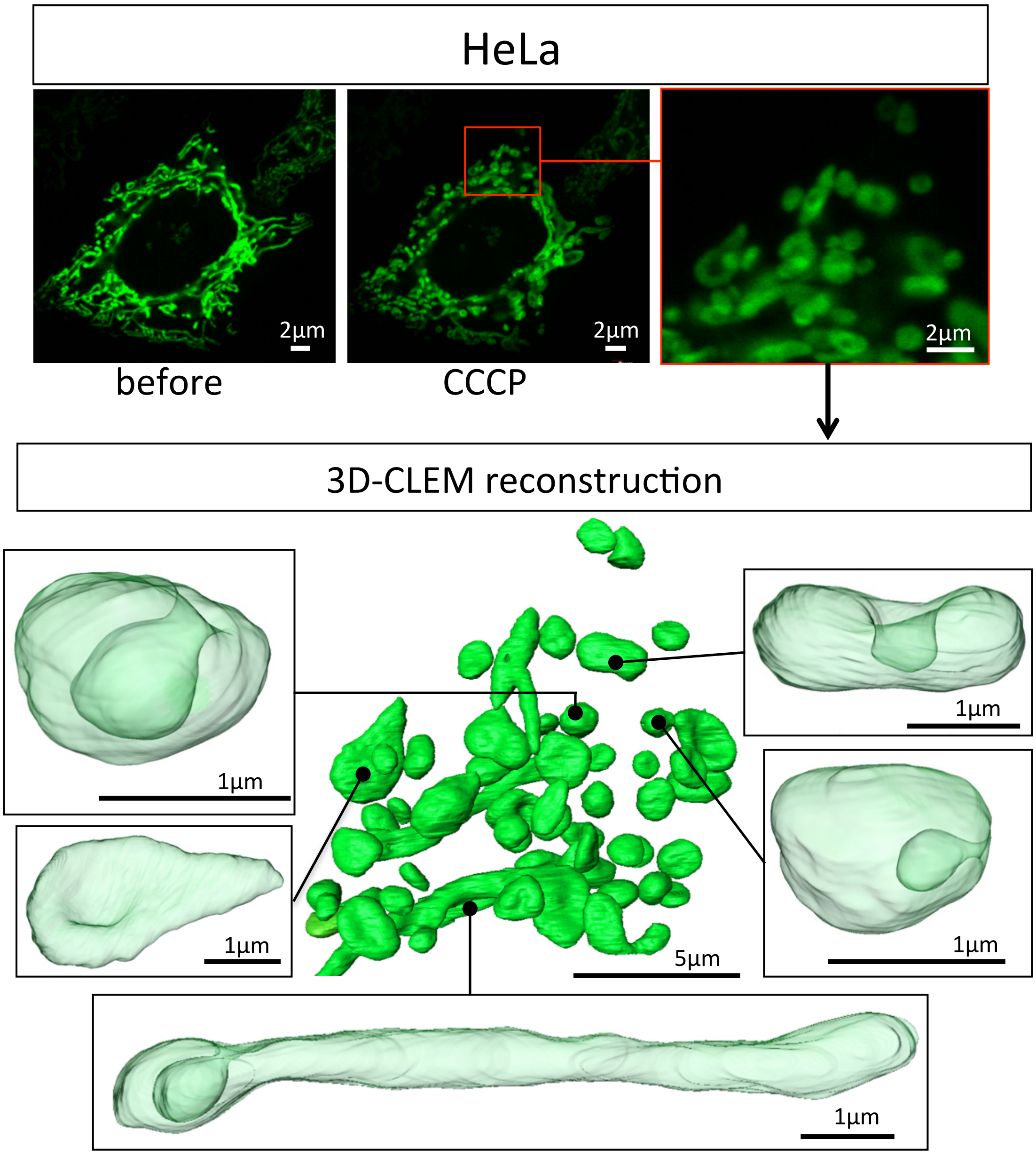

Supplementary Figure S5

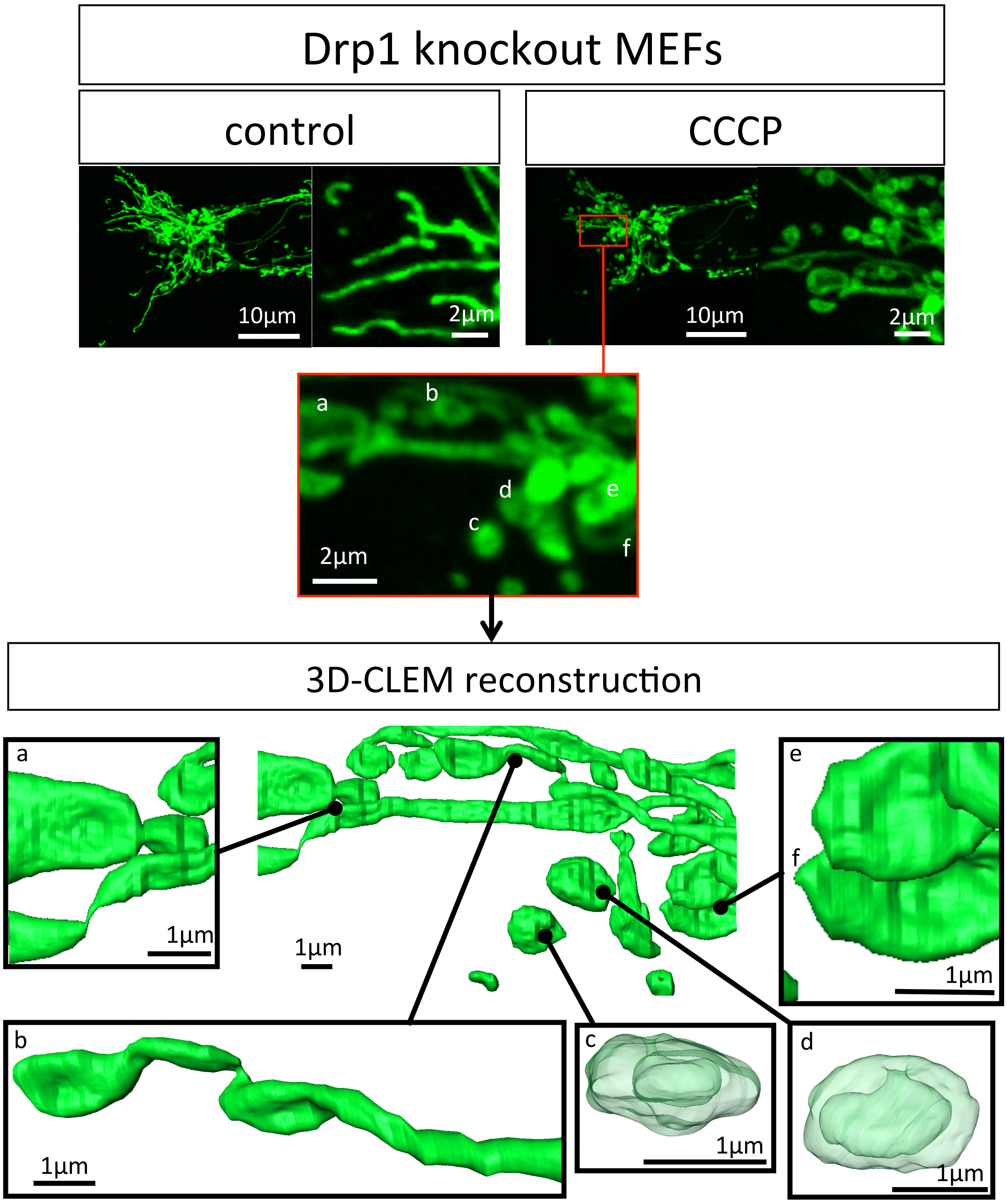

Supplementary Figure S6

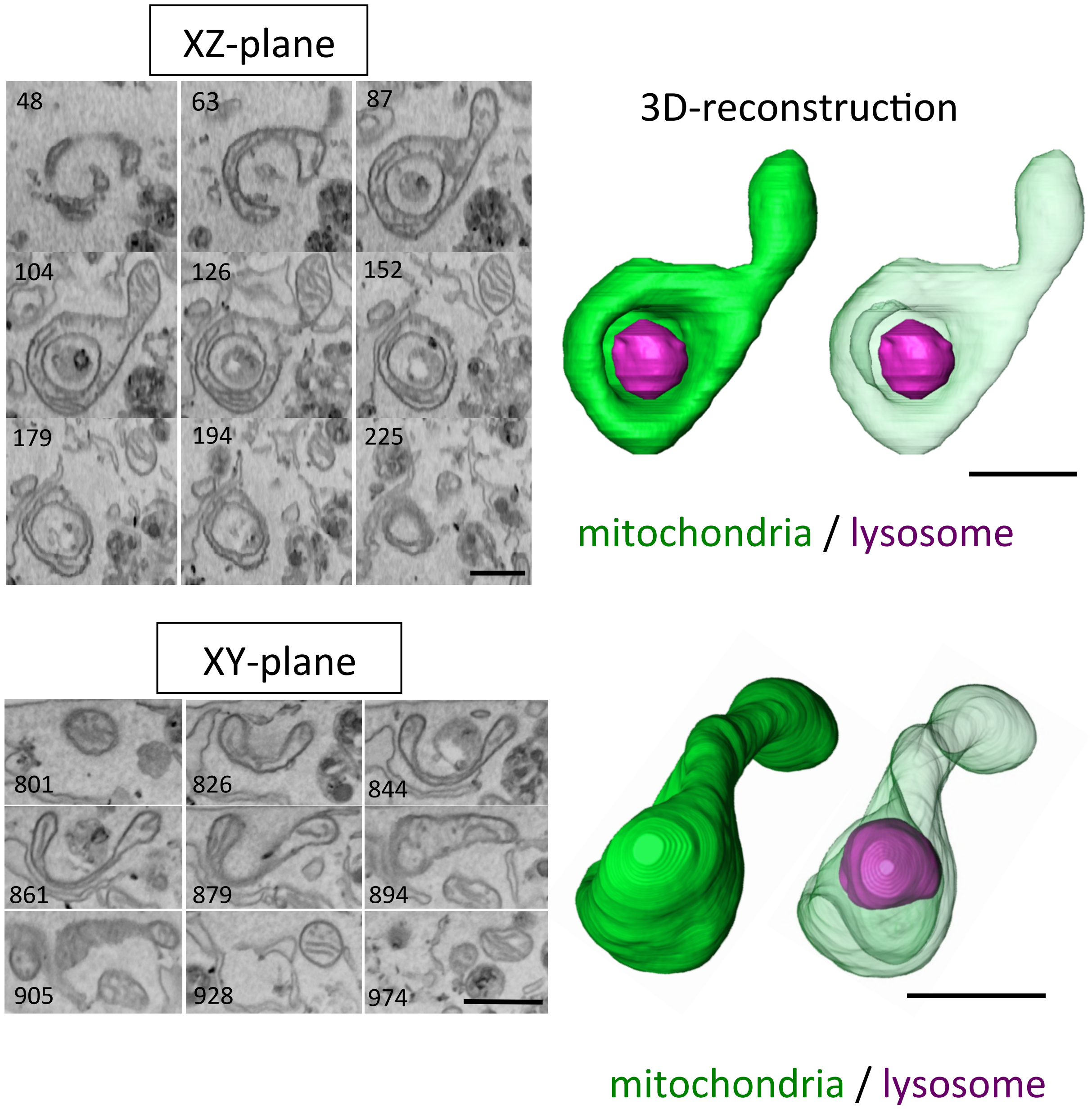

Supplementary Figure S7

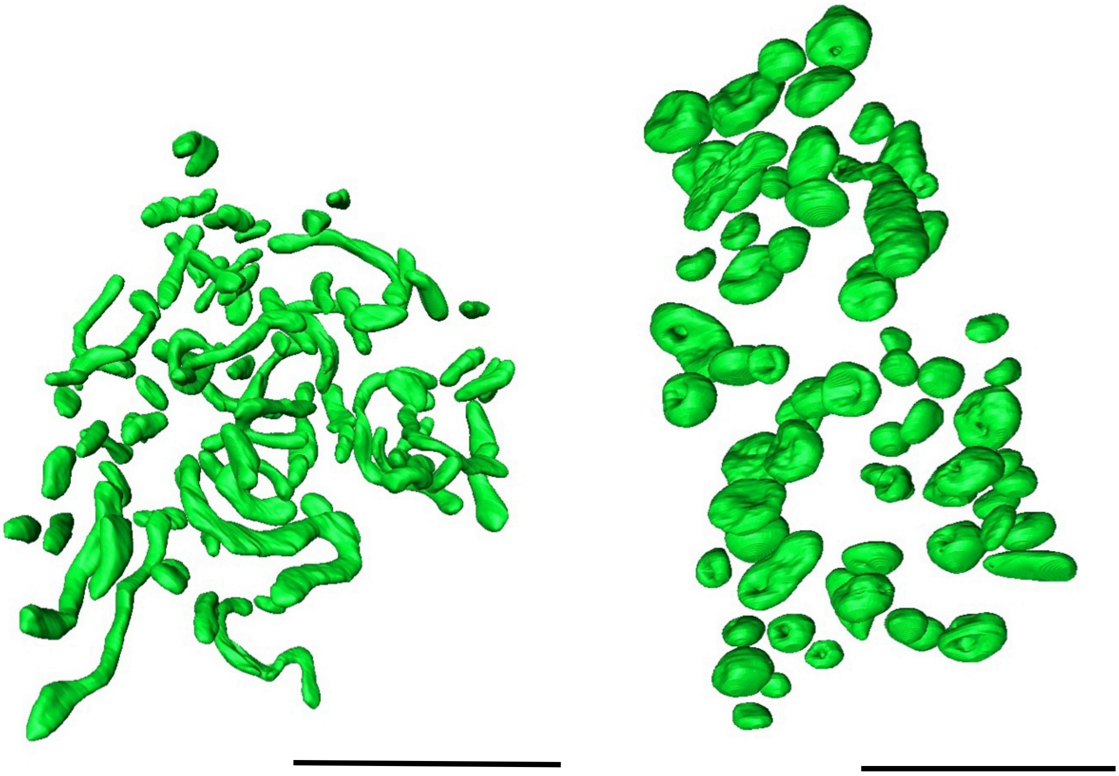

control

CCCP

Supplementary Figure S8

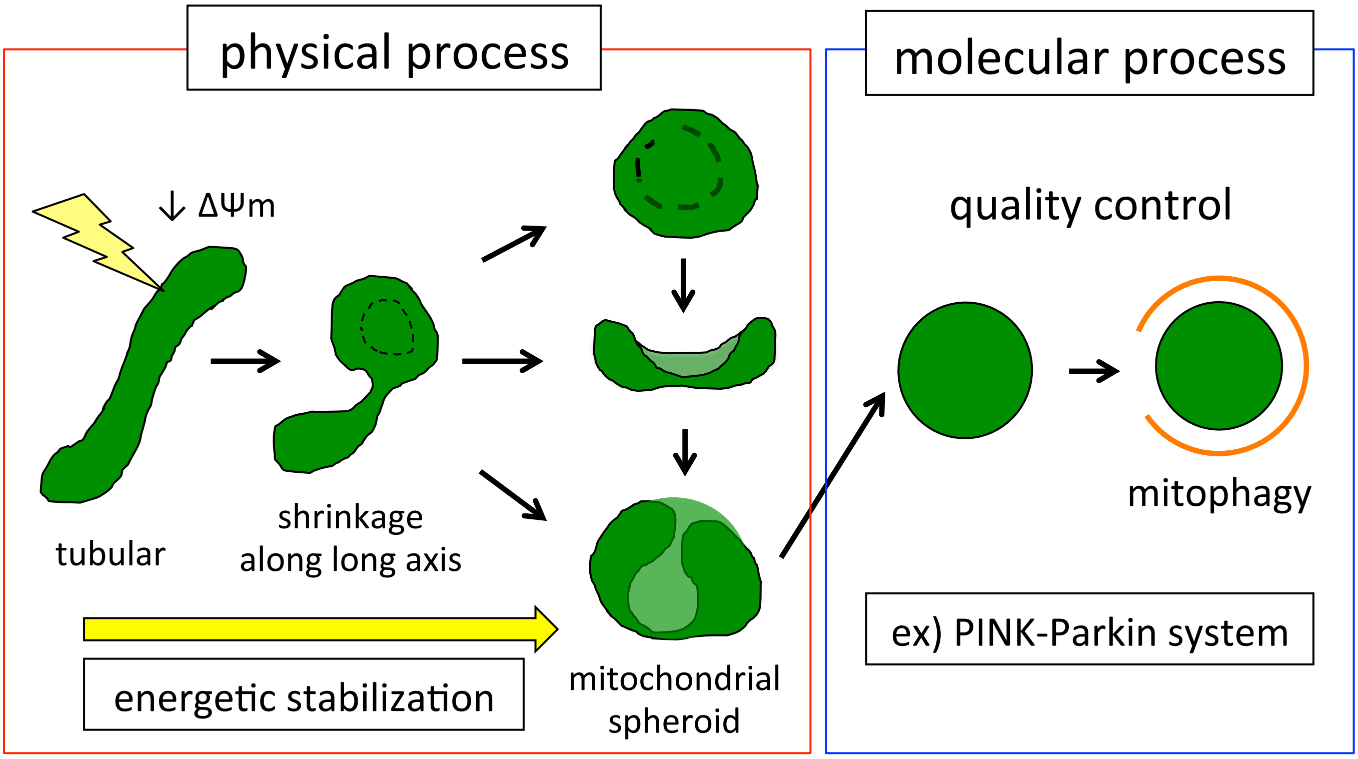

Supplement: Supplementary file 1 — supplementary figures and legends [file 41598_2017_18582_MOESM1_ESM.pdf]
